# Investigating antibody neutralization of lyssaviruses using lentiviral pseudotypes: a cross-species comparison

**DOI:** 10.1099/vir.0.2008/000349-0

**Published:** 2008-09

**Authors:** Edward Wright, Nigel J. Temperton, Denise A. Marston, Lorraine M. McElhinney, Anthony R. Fooks, Robin A. Weiss

**Affiliations:** 1 MRC/UCL Centre for Medical Molecular Virology, Division of Infection and Immunity, University College London, 46 Cleveland Street, London W1T 4JF, UK; 2 Rabies and Wildlife Zoonoses Group, Veterinary Laboratories Agency (Weybridge), WHO Collaborating Centre for the Characterisation of Rabies and Rabies-related Viruses, New Haw, Addlestone KT15 3NB, UK

## Abstract

Cross-neutralization between rabies virus (RABV) and two European bat lyssaviruses (EBLV-1 and -2) was analysed using lentiviral pseudotypes as antigen vectors. Glycoprotein (G-protein) cDNA from RABV challenge virus standard-11 (CVS-11) and EBLV-1 and -2 were cloned and co-expressed with human immunodeficiency virus (HIV) or murine leukemia virus (MLV) *gag*–*pol* and packageable green fluorescent protein (GFP) or luciferase reporter genes in human cells. The harvested lentiviral (HIV) vector infected over 40 % of baby hamster kidney (BHK) target cells, providing high-titre pseudotype stocks. Tests on blinded antibody-positive (*n*=15) and -negative (*n*=45) sera, predetermined by the fluorescent antibody virus neutralization (FAVN) test approved by the World Health Organization (WHO) and Office International des Epizooties (OIE), revealed that the CVS-11 pseudotype assay had 100 % concordance with FAVN and strongly correlated with neutralization titres (*r*^2^=0.89). Cross-neutralization tests using sera from RABV-vaccinated humans and animals on pseudotypes with CVS-11, EBLV-1 and EBLV-2 envelopes showed that the relative neutralization titres correlated broadly with the degree of G-protein diversity. Pseudotypes have three major advantages over live-virus neutralization tests: (i) they can be handled in low-biohazard-level laboratories; (ii) the use of reporter genes such as GFP or *β*-galactosidase will allow the assay to be undertaken at low cost in laboratories worldwide; (iii) each assay requires <10 μl serum. This robust microassay will improve our understanding of the protective humoral immunity that current rabies vaccines confer against emerging lyssaviruses, and will be applicable to surveillance studies, thus helping to control the spread of rabies.

## INTRODUCTION

Rabies virus (RABV) has the highest human case fatality rate of any viral disease, nearly 100 % in individuals who do not receive one of the available prophylaxes or post-exposure treatment. This high mortality rate coupled with the worldwide distribution of RABV means that approximately 55 000 people are killed each year, the main burden being borne by children in the developing world (WHO, 2006). There are effective pre- and post-exposure vaccines that are frontline mechanisms, along with the control of animal reservoirs, for combating the spread of the virus to susceptible animal hosts. These vaccines stimulate the production of virus-neutralizing antibodies (VNAs) to the envelope glycoprotein (G-protein), similar to the immunity conferred by live virus infections, where neutralizing immune responses to RABV are directed against the viral G-protein (Cox *et al.*, 1977). The host immune response also includes non-neutralizing antibodies and cell-mediated immunity to the nucleoprotein (Dietzschold *et al.*, 1987). Increased monitoring of humoral responses to RABV vaccines and studies of the prevalence of natural RABV and lyssavirus infections in domestic and wild species would allow for a more specific and proportionate response in endemic countries. However, the majority of these countries have resource-limited laboratories and the routine method to test for RABV VNAs, in most countries, involves high containment, biosafety level 3 facilities. Thus, it is currently not practical to undertake thorough and sustained surveillance as the screening cannot be undertaken in the majority of these locations.

European bat lyssavirus type-1 (EBLV-1) and European bat lyssavirus type-2 (EBLV-2) predominantly infect insectivorous bats. However, rare zoonotic events occur that have resulted in EBLV-1 infections of humans, sheep and a stone marten (Fooks *et al.*, 2003). Previous work has shown that antibody responses in individuals vaccinated with the RABV human diploid cell vaccine (HDCV) are able to neutralize RABV [rabies virus (RV61) and challenge virus standard-11 (CVS-11)], EBLV-1, EBLV-2 and Australian bat lyssavirus (Brookes *et al.*, 2005; Hanlon *et al.*, 2005). However, the same vaccine does not confer complete protection against the emerging Eurasian lyssavirus strains Aravan virus, Khujand virus, Irkut virus and West Caucasian Bat virus (Hanlon *et al.*, 2005) or against lyssaviruses belonging to phylogroup II (Badrane *et al.*, 2001; Fooks, 2004; Hanlon *et al.*, 2005).

Pseudotypes are viruses that carry the genome and core of one virus and the envelope of another. There are many examples of pseudotypes being used for virus receptor identification and cell entry (Dalgleish *et al.*, 1984; Wool-Lewis & Bates, 1998) and for virus neutralization by antibodies (Bartosch *et al.*, 2003; Clapham *et al.*, 1984; Temperton *et al.*, 2005). Retroviral pseudotypes [based on the gammaretrovirus murine leukemia virus (MLV) or lentivirus human immunodeficiency virus (HIV)] that bear vesicular stomatitis virus (VSV) G-proteins are commonly used as vectors for gene therapy (Naldini *et al.*, 1996; Takeuchi *et al.*, 1996; Zufferey *et al.*, 1997). In fact, retroviral pseudotypes using RABV envelopes have previously been shown to function as neurotropic gene vectors (Desmaris *et al.*, 2001; Mazarakis *et al.*, 2001; Mentis *et al.*, 2006). We have previously shown with SARS-coronavirus spike S-protein and H5N1 influenza virus haemagglutinin that retroviral pseudotypes expressing these envelope proteins can be used in sensitive and specific assays for the detection of VNAs to these pathogens (Temperton *et al.*, 2005, 2007).

Here we report the analysis of neutralizing antibodies to CVS-11, EBLV-1 and EBLV-2 in sera from RABV-vaccinated humans, canines and felines, using lentiviral pseudotypes. As the only lyssavirus protein present in the pseudotypes is the G-protein, we can accurately determine the precise role that antibodies targeting this protein play in cross-neutralization. Virus-neutralizing titres are correlated against full-length G-protein sequences encoding the glycoprotein. This powerful, novel assay for the detection of RABV VNA using CVS-11 pseudotypes can be adapted to meet the needs of many laboratories worldwide without requiring high containment. Using pseudotypes with the luciferase reporter, we established the assay's sensitivity and specificity with serum samples that were previously characterized using the World Health Organization (WHO) and Office International des Epizooties (OIE) fluorescent antibody virus neutralization test (FAVN).

## METHODS

### Cell lines.

Human embryonic kidney 293T cells (HEK-293T; originally referred to as 293/tsAJ609neo; DuBridge *et al.*, 1987) were used for transfections and to determine the tropism range of the pseudotyped virus together with the NP2 human glioma cell line (Soda *et al.*, 1999), baby hamster kidney 21 cells clone 13 (BHK-21; originally obtained from the American Tissue Culture Collection as ATCC CCL-10; Stoker & Macpherson, 1964), human rhabdomyosarcoma cells (TE671; Stratton *et al.*, 1989) and mouse neuroblastoma cells (N2A; originally obtained from ATCC CCL-131). HEK-293T cells were maintained in Dulbecco's modified Eagle medium (DMEM) supplemented with Glutamax and 15 % fetal calf serum (FCS) at 10 % CO_2_; all other cell lines were cultured in DMEM with 10 % FCS and 1 % penicillin/streptomycin at 5 % CO_2_.

### Plasmids and pseudotype production.

For transfection, 5×10^6^ HEK-293T cells were plated 24 h prior to addition of a complex comprising plasmid DNA and Fugene 6 that facilitated DNA transport into the cells (as described by the manufacturer; Roche). The HIV type 1 (HIV-1) *gag*-*pol* construct pCMV-Δ8.91 (Zufferey *et al.*, 1997) and green fluorescent protein (GFP) reporter construct pCSGW (pHR′SIN-cPPT-SGW, which incorporates the eGFP cassette driven by the U3 part of the spleen focus forming virus long-terminal repeat sequence; Demaison *et al.*, 2002) or the firefly luciferase reporter construct pCSFLW (where the luciferase gene has been cloned into pCSGW in place of GFP; a kind gift from Dr B. Capecchi, Novartis Vaccines and Diagnostics, Siena, Italy), were transfected concurrently with the required envelope construct at a ratio of 1 : 1.5 : 1 μg, respectively. MLV *gag*-*pol* construct pCMVi (Towers *et al.*, 2000) and GFP reporter construct pCNCG [a LNCX plasmid (CLONTECH) encoding enhanced GFP, with CMV driving expression of the RNA] or firefly luciferase reporter construct (Op De Beeck *et al.*, 2004) were used at the same ratio as the HIV constructs. In each case, cells were washed 24 h post-transfection and incubated with fresh media. Supernatants were harvested 48 and 72 h post-transfection and titrated on HEK-293T, NP2, BHK-21, TE671 and N2A cell lines. The remaining virus was stored at −80 °C. Fresh or frozen pseudotype aliquots were used for virus titrations and neutralization assays, respectively. Each round of freeze-thaw resulted in an average loss in virus titre of 5.7 % for CVS-11, 3 % for EBLV-1, 5.3 % for EBLV-2 and 2% for VSV pseudotype (see Supplementary Fig. S1, available with the online version of this paper).

### Viruses and envelope cloning.

The RABV isolate used in this study was CVS-11 (ATCC VR-959). Its G-gene sequence was amplified using the RT-PCR Titan kit as described by the manufacturer (Roche), with primers that were designed based on the published sequence (GenBank accession number EU352767). EBLV-1 (isolate RV 9) and EBLV-2 (isolate RV 1787) G-gene sequences (GenBank accession numbers EU352768 and EU352769, respectively) were subcloned from existing transfer plasmids using specific PCR primer sets. Primers are listed in Supplementary Table S1. The PCRs introduced unique *Kpn*I and *Xho*I restriction sites at the 5′ and 3′ ends of the genes. Once amplified, they were ligated into pI.18, a pUC-based plasmid incorporating the human cytomegalovirus immediate early promoter (Cox *et al.*, 2002), and sequenced to ensure correct alignment.

The VSV G-protein (Indiana serotype) expression vector pMD.G has been described previously (Naldini *et al.*, 1996) and was used to create a control pseudotype virus.

### Western blot.

CVS-11, ΔEnv (where only the plasmids containing *gag*-*pol* and reporter gene were transfected into cells), HIV and VSV pseudotyped viral particles were purified on a 25/40 % sucrose cushion to remove any G-protein not incorporated into the viral envelope before being separated by SDS-PAGE. Cell extracts for Western blots were prepared by resuspending 1×10^6^ HEK-293T cells (72 h post-transfection) in SDS-PAGE loading buffer (100 mM Tris/HCl, pH 6.8; 20 %, v/v, glycerol; 143 mM 2-mercaptoethanol; 10 %, v/v, SDS; and 0.025 %, w/v, bromophenol blue). Proteins were transferred, using semi-dry equipment, to a PVDF transfer membrane (Hybond-P; Amersham Biosciences) and blotted with SNB1, a primary mouse anti-RABV G-protein monoclonal antibody (1 : 500; a kind gift from Merial). Immunoblots for VSV G-protein were performed using an anti-VSV G-protein monoclonal antibody (clone P5D4; 1 : 10 000; Sigma). To determine protein loading/transfer, an identically loaded gel was stained with Coomassie blue and the nitrocellulose membrane was blotted with two anti-HIV-1 Gag p53/p24 antibodies (EVA365 and EVA366 diluted 1 : 100; AIDS Reagents, NIBSC) or an anti-actin antibody able to detect all isoforms (diluted 1 : 750; Sigma). For all blots, an anti-mouse horseradish peroxidase-conjugated IgG secondary antibody (diluted 1 : 5000; Amersham Biosciences) was then used prior to antibody binding detection by enhanced chemiluminescence (Amersham Biosciences).

### Serum samples.

Varied samples comprising sera from RABV-vaccinated humans, dogs and cats and the OIE standard reference dog serum diluted to 0.5 IU ml^−1^ with Stabilzyme (Surmodics Inc.) were tested. Detailed descriptions of each serum are given in Supplementary Table S2. To determine the stringency of the assay, a total of 60 serum samples were used; these were 48 dog (40/8, negative/positive; as initially determined by FAVN), nine cat (5/4) and three human (0/3) sera. An additional rabbit serum, raised against EBLV-2, was used for the cross-neutralization experiments. Sera were titrated using twofold serial dilutions to obtain the IC_100_. All experiments were undertaken at least in duplicate; if the titre varied by more than one doubling dilution it was repeated a third time and the geometric mean was recorded, in keeping with standard serological practice (Bresson *et al.*, 2006).

### Neutralization assays.

Live virus experiments were undertaken using the FAVN as described previously using CVS-11 (Brookes *et al.*, 2005; Cliquet *et al.*, 1998). Pseudotype TCID_50_ values were calculated using the end point method (Condit, 2001); the CVS-11 pseudotype was serially diluted in multiple replicates which resulted in infection of all replicates at low dilutions, no infection at high dilutions and infection in some but not all inoculations at intermediate dilutions. The dilution of virus that resulted in infection of 50 % of replicates could then be calculated.

In a 96-well plate, 90×TCID_50_ CVS-11 pseudotyped virus that resulted in an output of 8×10^5^ relative light units (RLU) was incubated with doubling dilutions of sera for 1 h at 37 % (5 % CO_2_) before the addition of 1×10^4^ BHK-21 cells. These were incubated for a further 48 h, after which 125 μl medium was removed and 75 μl Bright-Glo reagent (Promega) was added. Luciferase activity was detected 2.5 min later by reading the plates on a Glomax 96 microplate luminometer (Promega). End-point titres (which are equivalent to IC_100_ values) were chosen, as used in FAVN tests. Our initial experiments using GFP reporter proteins were analysed by fluorescence microscopy and by flow cytometry (FACScan; BD). For all results, background RLU (virus alone or ΔEnv) or GFP (cells alone) was deducted before analysis.

Cross-neutralization experiments were performed as above with the following mean pseudotype input (RLU): CVS-11, 5.4×10^5^; EBLV-1, 4.5×10^5^; EBLV-2, 4.2×10^5^.

## RESULTS

### Expression of RABV G-protein and production of pseudotype particles

**Fig. 1. f1:**
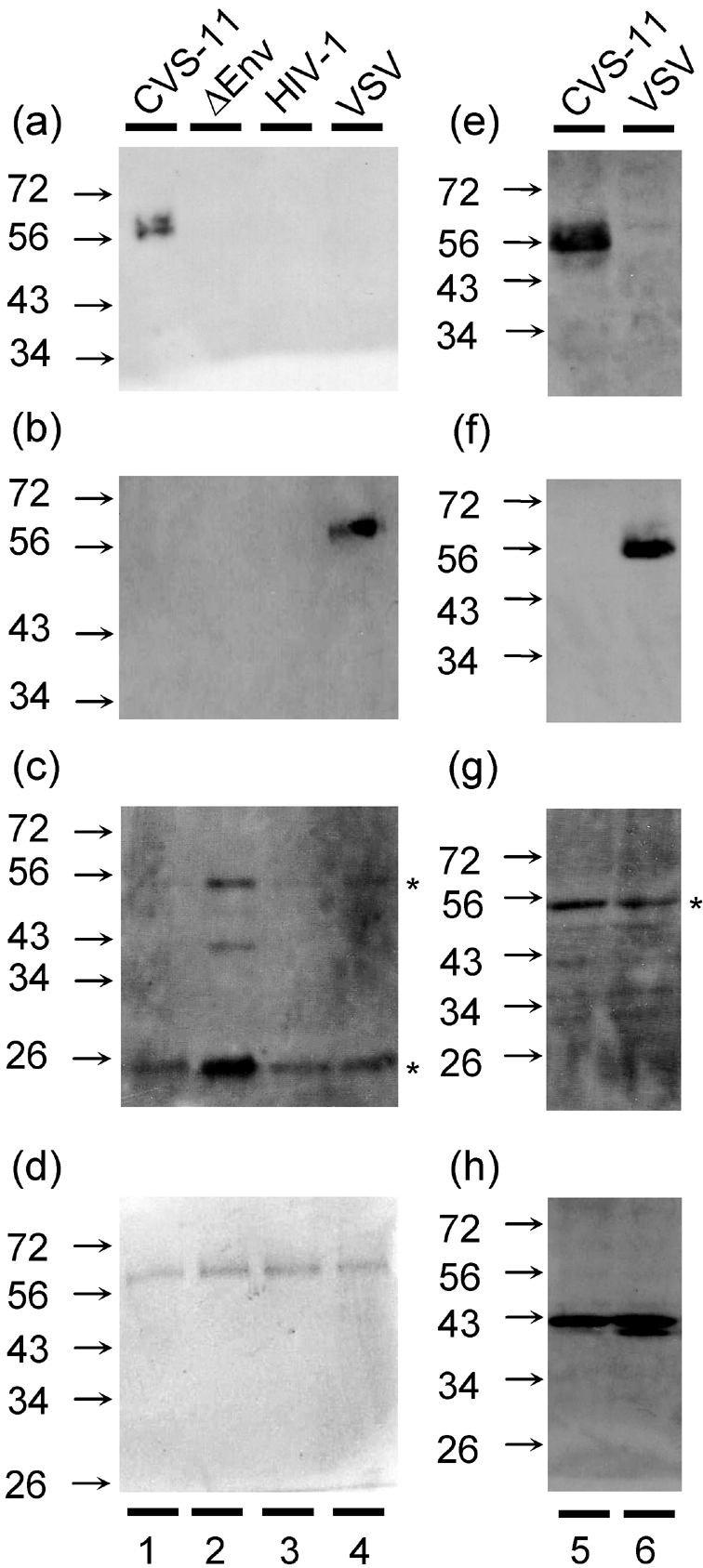
Expression of RABV-G, VSV-G and HIV-1 capsid proteins in pseudotypes and producer 293T cells. (a) An anti-RABV G-protein antibody was used in a Western blot of purified pseudotype samples with envelope glycolproteins of CVS-11 (lane 1), ΔEnv (lane 2), HIV (lane 3) and VSV (lane 4). (b) A comparative immunoblot for VSV was undertaken using an anti-VSV G-protein antibody. (c) Protein transfer was measured by blotting the membrane with antibodies to p53 and p24 of HIV-1. (d) An identical SDS gel was stained with Coomassie blue to determine total protein loading. Cells used to produce CVS-11 (lane 5) and VSV (lane 6) pseudotypes were harvested 72 h post-transfection and lysed in SDS-PAGE loading buffer. Extract equating to 4×10^4^ cells was separated by SDS-PAGE and the levels of CVS-11 G-protein (e), VSV G-protein (f), HIV-1 p53/24 (g) and actin (h) were determined using monoclonal antibodies. *, p53 and p24 staining.

To determine whether the viral G-proteins were being expressed on the envelope of the pseudotype virus, we performed a Western blot using supernatant from transfected cells and a RABV monoclonal antibody (SNB1). The viral particles were purified from cell supernatant using a sucrose cushion to ensure that no secreted envelope was present in the samples. We were able to detect RABV envelope protein in the CVS-11 sample only (lane 1; Fig. 1a[Fig f1]). No RABV G-protein was detected in the three negative control lanes (2–4) representing ΔEnv, HIV and VSV. To compare the level of CVS-11 G-protein expression on pseudotype particles with that of other viral envelopes, we blotted for VSV G-protein associated with a VSV pseudotype particle and observed similar levels of expression to CVS-11 (Fig. 1b[Fig f1]). Subsequent blotting of the membrane with anti-HIV-1 Gag p53/p24 antibodies confirmed the presence of lentiviral particles (Fig. 1c[Fig f1]). Protein loading was established by running an identical SDS gel and staining it with Coomassie blue (Fig. 1d[Fig f1]). We observed high, and comparable, expression of CVS-11 (Fig. 1e[Fig f1]), VSV G-protein (Fig. 1f[Fig f1]) and HIV-1 p53 (Fig. 1g[Fig f1]) when extracts from cells used to produce CVS-11 or VSV pseudotypes were analysed. An actin control was used to ensure equal protein loading (Fig. 1h[Fig f1]).

**Fig. 2. f2:**
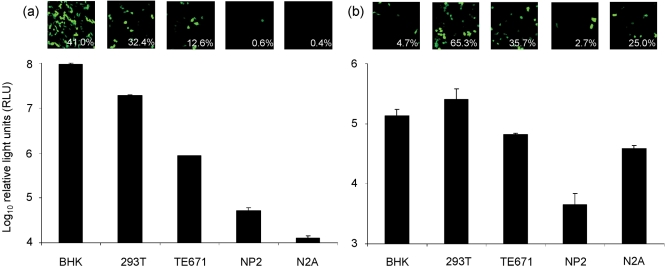
Cells permissive for the CVS-11 pseudotype virus. Aliquots of virus (50 μl) with a lentiviral (HIV; a) or a retroviral (MLV; b) core were incubated with different cell lines to determine the most appropriate cell type to use in further assays. GFP results are shown as fluorescence micrographs and mean proportions of infected cells (determined by flow cytometry). Viral titres for luciferase pseudotypes are given in RLU (mean±sd) and titres reported are after deduction of the background luciferase activity of ΔEnv pseudotypes observed on each cell line.

CVS-11 pseudotype virus preparations were made by packaging either GFP or firefly luciferase as a reporter gene and marker of infection. These were titrated on cell lines known to be permissive for infection with wild-type RABV, i.e. BHK and N2A cells, and others that had previously not been tested: 293T, TE671 and NP2. Luciferase-pseudotyped virus with an HIV (lentiviral) core produced up to 2.5 log_10_ higher viral titres (Fig. 2a[Fig f2]) than an MLV (gammaretroviral) core (Fig. 2b[Fig f2]). Conversely, GFP-MLV virus (Fig. 2b[Fig f2]) was more potent than GFP-HIV (Fig. 2a[Fig f2]), except on BHK cells. The 293T and TE671 cell lines were highly permissive for both the GFP and luciferase CVS-11 pseudotypes, but BHKs gave the strongest results for HIV-based pseudotypes, and the highest luciferase titre overall. NP2 cells appeared to be almost entirely refractory to infection, while N2As were only permissive for MLV-based pseudotypes. All the results for luciferase and GFP virus across the different cell lines correlated, with the exception of the MLV virus stocks on BHK cells. These results indicate that the HIV vector titrated on BHK cells yielded the most sensitive system for CVS-11 pseudotypes. We recorded an approximate GFP pseudotype titre of 1.3×10^5^ IU ml^−1^ for CVS-11 pseudotypes and 6.5×10^6^ IU ml^−1^ for pseudotype virus with the VSV G-protein. Since BHK cells are also used for FAVN assays, it allowed a direct comparison of neutralization as described below.

### Sensitivity of the neutralization assay using a CVS-11 pseudotype

In order to determine whether incubating varying amounts of pseudotype with antibody affected IC_100_ titres, increasing logs of pseudotypes were used in neutralization assays with the OIE reference standard, plus medium-titre (RC-199) and high-titre (RC-195) sera. Virus concentration was determined by TCID_50_ and by luciferase RLU. Using an input of 1000×TCID_50_ (1×10^7^ RLU), the OIE standard positive control (0.5 IU ml^−1^) could not achieve complete neutralization (Table 1). However, when the input was decreased to 100×TCID_50_ (1×10^6^ RLU) and then 10×TCID_50_ (1×10^5^ RLU), the OIE standard was able to neutralize 100 % at greater dilutions. The same effect was observed using sera from a feline (RC-195) and another canine (RC-199) sample. Thus, as the level of virus/antigen input increased, there was a concomitant decrease in the VNA titre (Table 1).

**Fig. 3. f3:**
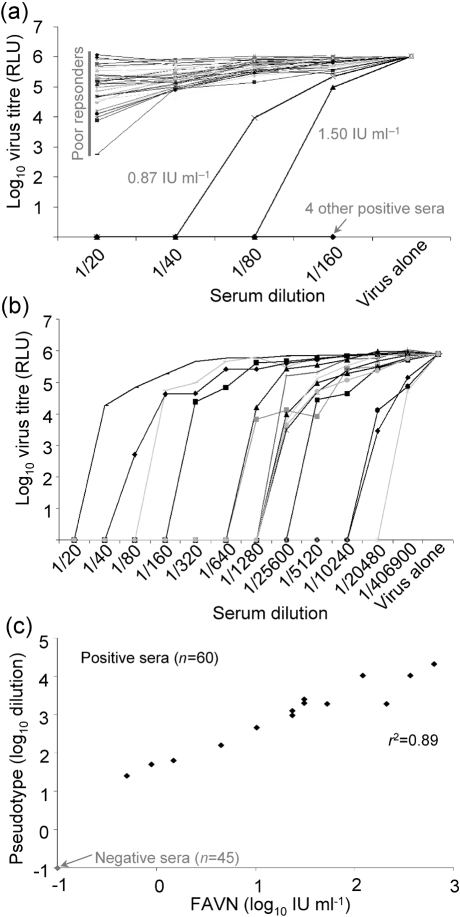
Comparison of neutralizing titres using FAVN and RABV lentiviral pseudotype reveals high specificity and sensitivity. (a) To determine the specificity of the assay, 50 blinded serum samples were tested. All 44 negative and six positive samples (by FAVN) were identified correctly. (b) Fifteen positive samples were subsequently tested to determine the sensitivity of the neutralization assay using lentiviral pseudotypes. (c) The results obtained for each sample tested were plotted on a scatter plot showing the correlation between FAVN and pseudotype results given in IU ml^−1^ and dilution respectively. The position of negative sera is shown in light print; two positive samples have the same IU and dilution values, so appear as one point.

Having established the working concentration of the luciferase pseudotype virus, we tested 50 blinded sera from RABV vaccine recipients to an end point of four doubling dilutions initially, to determine which samples were the negatives or failures (<0.5 IU ml^−1^; Fig. 3a[Fig f3]). There were 44 of these, with poor or no response that gave insufficient protection according to OIE/WHO criteria, and six clearly positive sera with differing titres. The six positive sera were tested at a higher serum dilution in tandem with a further nine positive samples, blinded with respect to their FAVN neutralization titres (Fig. 3b[Fig f3]). When unblinded, these results mirrored those obtained using the FAVN test. A panel of low-, medium- and high-titre RABV-specific sera failed to neutralize pseudotypes bearing the VSV G-protein (data not shown).

To determine how sensitive the pseudotype assay was at differentiating between serum samples with different neutralizing titres, we compared the titres obtained using the pseudotype assay with FAVN titres. The two sets of titres correlated strongly, with those sera that had a high IU ml^−1^ reading also scoring a higher dilution result (Table 2). These data also showed that different animal species (feline, canine, human) elicited a range of strong and weak VNA responses to RABV. Taken together and presented as a scatter plot, the results obtained revealed a high correlation coefficient (*r*^2^=0.89) between pseudotype and FAVN titres (Fig. 3c[Fig f3]).

### Cross-neutralization of CVS-11 and European bat lyssaviruses

Having established a working protocol for the accurate determination of CVS-11 virus-neutralizing antibodies, we adapted the assay to detect humoral responses to pseudotypes bearing G-proteins from representative isolates of EBLV-1 and EBLV-2. We could then study what effect antibodies that specifically target the G-protein alone have on cross-neutralization, and whether any animal species elicit a more potent response than others.

**Fig. 4. f4:**
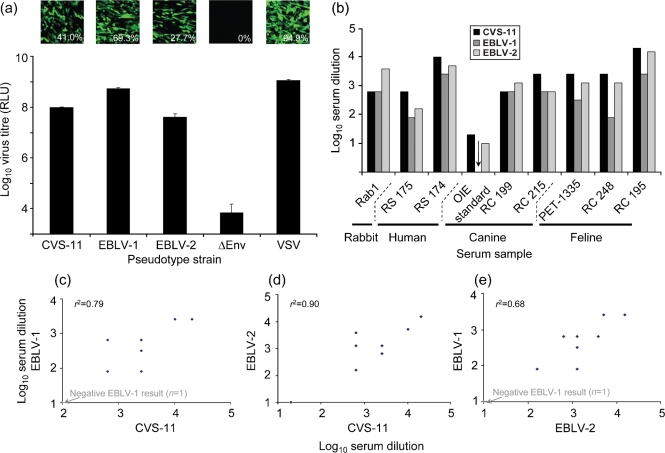
Cross-neutralization of RABV and bat lyssaviruses by rabbit, human, canine and feline sera. (a) EBLV-1 and EBLV-2 pseudotypes with GFP and luciferase reporters (50 μl) were tested on BHK cells alongside CVS-11, ΔEnv and VSV controls. (b) IC_100_ VNA titres for rabbit, human, canine and feline sera are reported for CVS-11, EBLV-1 and EBLV-2 luciferase pseudotypes and scatter plots with correlation co-efficients comparing CVS-11 and EBLV-1 (c), CVS-11 and EBLV-2 (d) and EBLV-1 and EBLV-2 (e) are given. The position of negative sera is shown in light print (c, e); two positive samples in (c) and (d) had the same serum dilution value for both viruses, so they appear as one point. For (c), (d) and (e), *n*=9.

The titre of pseudotype virus obtained with EBLV-1 and EBLV-2 G-protein pseudotypes was comparable to that for CVS-11 and within a log_10_ of the titre achieved with VSV G-protein (Fig. 4a[Fig f4]).

We therefore compared the patterns observed for VNA responses of RABV antisera from different animals with different lyssaviruses (Fig. 4b[Fig f4]). Human serum samples neutralized CVS-11 to the greatest degree, followed by EBLV-2 and then EBLV-1; the same neutralizing profile was observed in cats. The sera from dogs neutralized all three genotypes, but no obvious pattern was common to these samples. Antibodies in the rabbit serum also neutralized all three pseudotypes, with the greatest neutralization observed with EBLV-2. When all nine neutralizing responses against CVS-11 and EBLV-1 were compared (Fig. 4c[Fig f4]), there was a correlation coefficient of 0.79, while that between CVS-11 and EBLV-2 was 0.90 (Fig. 4d[Fig f4]) and between EBLV-1 and EBLV-2 was 0.68 (Fig. 4e[Fig f4]). Analysis of the full-length G-protein amino acid sequences used here revealed that they have a similar degree of divergence from each other, with EBLV-1 and EBLV-2 (80 %) being marginally more closely related to each other than to CVS-11 (70.1 and 73.7 % respectively; Table 3). The *r*^2^ values for CVS-11 versus EBLV-1 and CVS-11 versus EBLV-2 rank in inverse order to the degree of amino acid diversity of their respective G-proteins.

## DISCUSSION

Our pseudotype particles that express RABV envelope/G-proteins are antigenically similar to those native proteins on wild-type, live RABV virus particles, which function to allow particle entry and are neutralized by anti-RABV sera. Using an antibody shown previously to detect RABV G-protein, we were able to detect these proteins in immunoblots conducted with our CVS-11 pseudotype virus. This antibody does not detect G-protein in ΔEnv virus (lacking a RABV envelope glycoprotein) or on pseudotypes bearing either the HIV envelope or VSV G-protein, although VSV and RABV are members of the same virus family, *Rhabdoviridae*. However, using a VSV-G monoclonal antibody, we observed the same, if not marginally less, incorporation of the envelope protein into the pseudotype. The levels of CVS-11 and VSV G-protein expression are also comparable in the transfected 293T producer cells, suggesting that the G-proteins incorporate with equal efficiency into the pseudotypes. As expected, the cleaved Gag p24 protein predominates in the mature pseudotype particle and the unspliced Gag p53 predominates in the 293T cells.

The use of replication-competent isolates of highly pathogenic viruses is strictly regulated and, in the majority of countries, requires the use of category 3 or 4 containment laboratories. Using pseudotype technology, we have constructed modified lentiviral vectors and established an assay that utilizes these replication-incompetent particles to measure RABV VNA titres accurately. This method has the benefit of allowing experiments to be undertaken in category 2 biosafety laboratories, since the pseudotype is unable to replicate and cause a productive infection. The assay also benefits from detecting VNA alone, in contrast to ELISA, but similar to FAVN, gives a more accurate picture of the protective antibodies present in the sample. Over 60 sera from various host species were evaluated for neutralizing antibodies against the CVS-11 G-protein. The results show that the pseudotype assay is 100 % specific and equally sensitive compared with the WHO/OIE-approved FAVN assay, as shown by effective differentiation between positive and negative sera and by accurately reflecting VNA titres for the positive sera.

It has been reported that human sera from recipients of the RABV HDCV can cross-neutralize EBLV-1 and EBLV-2 (Brookes *et al.*, 2005; Hanlon *et al.*, 2005). Confirmation of these observations using pseudotypes is consistent with the notion that G-protein is the target for neutralizing antibody because it is the only lyssavirus protein present in the pseudotype particle. Furthermore, our results show that antibodies in serum from vaccinated animals, specifically targeting the G-protein, cross-neutralize distinct lyssavirus genotypes to varying titres depending on viral genotype and the animal that the serum was obtained from. G-protein antibody responses in humans and cats were higher against CVS-11 infections, followed by EBLV-2 and finally EBLV-1, but additional samples need testing to confirm this observation. This result is to be expected to some extent, given that the samples were from RABV-vaccinated individuals. As expected, the rabbit serum, which was raised against EBLV-2, neutralized this genotype at higher dilutions than CVS-11 and EBLV-1.

Our cross-neutralization data for CVS-11 versus EBLV-1 or EBLV-2 support the hypothesis that the higher the degree of similarity between G-protein sequences the more comparable the neutralization titres (Brookes *et al.*, 2005; Hanlon *et al.*, 2005). The lower *r*^2^ value for EBLV-1 versus EBLV-2 does not support this hypothesis, however, as the EBLVs have a higher sequence identity to each other than to CVS-11. It is likely that this is due to all but one of the sera used in these experiments being raised only against RABV-based vaccines. In addition, the small sample size and individual variation may influence the results. This illustrates the difficulties in interpretation of antigenic differences using cross-neutralization data. A wider study using multiple representatives from each genotype, with sera raised against multiple genotypes, may assist in further quantifying antigenic differences between lyssaviruses.

The cross-neutralization of lentiviral pseudotypes bearing lyssavirus envelopes will provide the opportunity to analyse neutralizing epitopes in G-proteins quantitatively and qualitatively. The method described here can be extended to other lyssaviruses in phylogroups 1 and 2. Because cDNA cloned into plasmids is used to generate the pseudotype, site-specific mutagenesis can be exploited to elucidate which domains are crucial for G-protein function in effecting virion attachment and entry, and which neutralizing epitopes determine strain-specific and group-specific responses to sera and to monoclonal antibodies.

For the adoption of neutralization assays, using either replication-competent or pseudotype viruses, there are some issues that need to be addressed before the assay can be employed for routine serological surveillance. In both systems, there will be free or secreted envelope protein in the virus preparations that can adsorb antibody from the supernatant and thus skew neutralization tests. It is therefore important to standardize virus input in order to obtain the most sensitive and reliable results. This can be achieved by determining the concentration of virus at which a known negative serum becomes positive, and by using a concentration of virus slightly greater than that routinely used by rabies reference laboratories as part of their assay validation process. Alternatively, where a baseline positive control is available, for example the OIE reference standard for RABV neutralization assays, the smallest amount of virus that is needed to achieve an IC_100_ at the lowest serum dilution could be determined. This will allow for the greatest range of serum dilutions to be tested, while all negative samples will provide a ‘failed’ result. For our assay, this result was achieved using a virus input between 10×TCID_50_ (1×10^5^ RLU) and 100×TCID_50_ (1×10^6^ RLU). Therefore, we would recommend 10–100×TCID_50_ or 1×10^5^–1×10^6^ RLU virus input for neutralization assays using the CVS-11 pseudotype. While background readings for cells alone or virus alone were 50–200 RLU, luciferase carry-over for ΔEnv samples and those pseudotypes that produced a negative GFP result was up to 1×10^4^ RLU, depending on the cell line used (with 50 μl pseudotype input). Therefore, the ΔEnv value should be considered the cut-off for a positive viral titre when using large volumes of pseudotype. However, as only 0.1–0.5 μl of pseudotype virus is normally used, the ‘virus alone’ luciferase reading is generally larger than that which would be observed with ΔEnv and is therefore used as the background value.

It was encouraging to observe that BHK cells, which are routinely used in live virus CVS-11 FAVN tests, were highly permissive for our CVS-11 pseudotype. Conversely, N2A cells that were readily infected by live lyssaviruses were not permissive for the CVS-11 pseudotype with HIV core. In contrast, MLV-based pseudotypes, made using different reporter constructs, were able to infect N2A cells, suggesting a post-entry restriction imposed selectively on the lentiviral reporter gene in the vector. This could be due to a specific restriction of the pCSGW and pCSFLW promoter in this cell line, because N2A cells were also almost completely refractory to infection by VSV, EBLV-1 and EBLV-2 pseudotypes produced with the same HIV reporter plasmids (data not shown). Alternatively, the lentiviral core may be subject to a post-penetration restriction in N2A cells, such as TRIM5*α* (Towers, 2007).

The fatality rate of RABV infections in humans who have not received either pre- or post-exposure prophylaxis is very high, nearing 100 %. Compared with the diseases caused by other highly pathogenic viruses, such as Ebola virus haemorrhagic fever (80 % fatality) and H5N1 influenza (61 %), the ongoing research and surveillance for RABV is limited, supporting the suggestion that rabies is a ‘neglected disease’, especially in rabies-endemic countries in Africa and Asia (WHO, 2006; Fooks, 2005). There is, therefore, a need to readdress this balance by increasing the current level of surveillance for RABV in countries where the infection causes high levels of mortality. This would also help to limit misdiagnosis and consequent underreporting of RABV infection (Mallewa *et al.*, 2007). The main assay currently being used (FAVN) provides good sensitivity and specificity, but requires handling of live RABV. Alternative assays for the detection of RABV antibodies, such as ELISA (Cliquet *et al.*, 2004) and rapid fluorescent focus inhibition test-GFP (Khawplod *et al.*, 2005), have been designed; however, the former does not differentiate between neutralizing and non-neutralizing antibodies and the latter still requires high containment-level facilities.

The use of pseudotypes for the detection of VNAs removes the need to use live viruses and provides both high sensitivity and high specificity for the detection of neutralizing antibodies. Incorporation of GFP or luciferase as a reporter gene makes this assay applicable to many laboratories involved in RABV surveillance, and a good candidate for high throughput screening. For laboratories lacking fluorescence or luciferase detection, a *β*-galactosidase reporter could be used. It is also amenable to testing for the presence of antibodies in small volumes of sera (microassay) such as may be obtained from bats. In conclusion, this report shows that it is possible to analyse cross-neutralizing antibody responses against different lyssavirus genotypes using lentiviral pseudotypes. This approach could greatly improve the surveillance of new and emerging lyssaviruses and evaluation of the protection that current vaccines offer against these pathogens.

## Supplementary Material

[Supplementary Material]

## Figures and Tables

**Table 1. t1:** Effect of varying levels of pseudotype input on neutralization titres Sera were classified as low, medium or high by their FAVN titre

**Sera**	**Pseudotype virus input (RLU/TCID_50_)**		
	**1×10^5^/10**	**1×10^6^/100**	**1×10^7^/1000**
Low (0.5 IU ml^−1^; OIE standard)	40	20	<20
Medium (23.4 IU ml^−1^; RC-199)	2 560	1 280	320
High (364.5 IU ml^−1^; RC-195)	20 480	10 240	320

**Table 2. t2:**
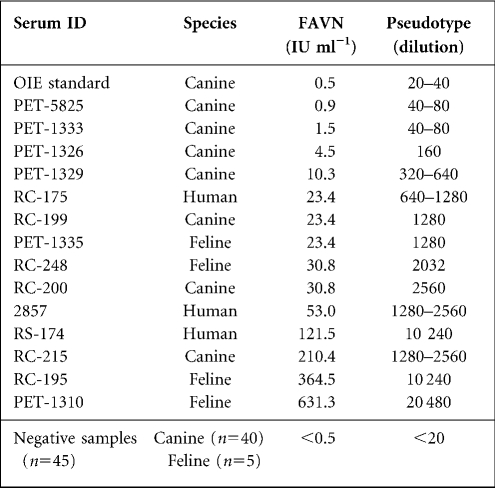
FAVN and pseudotype neutralizing titres for all serum samples

**Table 3. t3:** G-protein nucleotide and amino acid sequence identities for CVS-11, EBLV-1 and EBLV-2 Values above the diagonal refer to nucleotide identities; values below the diagonal refer to amino acid identities

**Virus**	**CVS-11**	**EBLV-1**	**EBLV-2**
**CVS-11**		66.3	69.8
**EBLV-1**	70.1		73.2
**EBLV-2**	73.7	80.0	

## References

[r1] Badrane, H., Bahloul, C., Perrin, P. & Tordo, N. (2001). Evidence of two Lyssavirus phylogroups with distinct pathogenicity and immunogenicity. J Virol 75, 3268–3276. 11238853 10.1128/JVI.75.7.3268-3276.2001 PMC114120

[r2] Bartosch, B., Bukh, J., Meunier, J. C., Granier, C., Engle, R. E., Blackwelder, W. C., Emerson, S. U., Cosset, F. L. & Purcell, R. H. (2003). *In vitro* assay for neutralizing antibody to hepatitis C virus: evidence for broadly conserved neutralization epitopes. Proc Natl Acad Sci U S A 100, 14199–14204. 14617769 10.1073/pnas.2335981100 PMC283569

[r3] Bresson, J. L., Perronne, C., Launay, O., Gerdil, C., Saville, M., Wood, J., Hoschler, K. & Zambon, M. C. (2006). Safety and immunogenicity of an inactivated split-virion influenza A/Vietnam/1194/2004 (H5N1) vaccine: phase I randomised trial. Lancet 367, 1657–1664. 16714186 10.1016/S0140-6736(06)68656-X

[r4] Brookes, S. M., Parsons, G., Johnson, N., McElhinney, L. M. & Fooks, A. R. (2005). Rabies human diploid cell vaccine elicits cross-neutralising and cross-protecting immune responses against European and Australian bat lyssaviruses. Vaccine 23, 4101–4109. 15964478 10.1016/j.vaccine.2005.03.037

[r5] Clapham, P., Nagy, K. & Weiss, R. A. (1984). Pseudotypes of human T-cell leukemia virus types 1 and 2: neutralization by patients' sera. Proc Natl Acad Sci U S A 81, 2886–2889. 6326149 10.1073/pnas.81.9.2886 PMC345177

[r6] Cliquet, F., Aubert, M. & Sagne, L. (1998). Development of a fluorescent antibody virus neutralisation test (FAVN test) for the quantitation of rabies-neutralising antibody. J Immunol Methods 212, 79–87. 9671155 10.1016/s0022-1759(97)00212-3

[r7] Cliquet, F., McElhinney, L. M., Servat, A., Boucher, J. M., Lowings, J. P., Goddard, T., Mansfield, K. L. & Fooks, A. R. (2004). Development of a qualitative indirect ELISA for the measurement of rabies virus-specific antibodies from vaccinated dogs and cats. J Virol Methods 117, 1–8. 15019254 10.1016/j.jviromet.2003.12.001

[r8] Condit, R. C. (2001). Principles of Virology. In *Fields Virology*, 4th edn, vol. 1, pp. 19–51. Edited by D. M. Knipe & P. M. Howley. Philadelphia: Lippincott Williams & Wilkins.

[r9] Cox, J. H., Dietzschold, B. & Schneider, L. G. (1977). Rabies virus glycoprotein. II. Biological and serological characterization. Infect Immun 16, 754–759. 408269 10.1128/iai.16.3.754-759.1977 PMC421026

[r10] Cox, R. J., Mykkeltvedt, E., Robertson, J. & Haaheim, L. R. (2002). Non-lethal viral challenge of influenza haemagglutinin and nucleoprotein DNA vaccinated mice results in reduced viral replication. Scand J Immunol 55, 14–23. 11841688 10.1046/j.1365-3083.2002.01015.x

[r11] Dalgleish, A. G., Beverley, P. C., Clapham, P. R., Crawford, D. H., Greaves, M. F. & Weiss, R. A. (1984). The CD4 (T4) antigen is an essential component of the receptor for the AIDS retrovirus. Nature 312, 763–767. 6096719 10.1038/312763a0

[r12] Demaison, C., Parsley, K., Brouns, G., Scherr, M., Battmer, K., Kinnon, C., Grez, M. & Thrasher, A. J. (2002). High-level transduction and gene expression in hematopoietic repopulating cells using a human immunodeficiency virus type 1-based lentiviral vector containing an internal spleen focus forming virus promoter. Hum Gene Ther 13, 803–813. 11975847 10.1089/10430340252898984

[r13] Desmaris, N., Bosch, A., Salaun, C., Petit, C., Prevost, M. C., Tordo, N., Perrin, P., Schwartz, O., de Rocquigny, H. & other authors (2001). Production and neurotropism of lentivirus vectors pseudotyped with lyssavirus envelope glycoproteins. Mol Ther 4, 149–156. 11482987 10.1006/mthe.2001.0431

[r14] Dietzschold, B., Lafon, M., Wang, H., Otvos, L., Jr, Celis, E., Wunner, W. H. & Koprowski, H. (1987). Localization and immunological characterization of antigenic domains of the rabies virus internal N and NS proteins. Virus Res 8, 103–125. 2445121 10.1016/0168-1702(87)90023-2

[r15] DuBridge, R. B., Tang, P., Hsia, H. C., Leong, P. M., Miller, J. H. & Calos, M. P. (1987). Analysis of mutation in human cells by using an Epstein-Barr virus shuttle system. Mol Cell Biol 7, 379–387. 3031469 10.1128/mcb.7.1.379-387.1987 PMC365079

[r16] Fooks, A. (2004). The challenge of new and emerging lyssaviruses. Expert Rev Vaccines 3, 333–336. 15270628 10.1586/14760584.3.4.333

[r17] Fooks, A. R. (2005). Rabies remains a ‘neglected disease’. Euro Surveill 10, 211–212. 16972345

[r18] Fooks, A. R., Brookes, S. M., Johnson, N., McElhinney, L. M. & Hutson, A. M. (2003). European bat lyssaviruses: an emerging zoonosis. Epidemiol Infect 131, 1029–1039. 14959767 10.1017/s0950268803001481 PMC2870049

[r19] Hanlon, C. A., Kuzmin, I. V., Blanton, J. D., Weldon, W. C., Manangan, J. S. & Rupprecht, C. E. (2005). Efficacy of rabies biologics against new lyssaviruses from Eurasia. Virus Res 111, 44–54. 15896401 10.1016/j.virusres.2005.03.009

[r20] Khawplod, P., Inoue, K., Shoji, Y., Wilde, H., Ubol, S., Nishizono, A., Kurane, I. & Morimoto, K. (2005). A novel rapid fluorescent focus inhibition test for rabies virus using a recombinant rabies virus visualizing a green fluorescent protein. J Virol Methods 125, 35–40. 15737414 10.1016/j.jviromet.2004.12.003

[r21] Mallewa, M., Fooks, A. R., Banda, D., Chikungwa, P., Mankhambo, L., Molyneux, E., Molyneux, M. E. & Solomon, T. (2007). Rabies encephalitis in malaria-endemic area, Malawi, Africa. Emerg Infect Dis 13, 136–139. 17370529 10.3201/eid1301.060810 PMC2725806

[r22] Mazarakis, N. D., Azzouz, M., Rohll, J. B., Ellard, F. M., Wilkes, F. J., Olsen, A. L., Carter, E. E., Barber, R. D., Baban, D. F. & other authors (2001). Rabies virus glycoprotein pseudotyping of lentiviral vectors enables retrograde axonal transport and access to the nervous system after peripheral delivery. Hum Mol Genet 10, 2109–2121. 11590128 10.1093/hmg/10.19.2109

[r23] Mentis, G. Z., Gravell, M., Hamilton, R., Shneider, N. A., O'Donovan, M. J. & Schubert, M. (2006). Transduction of motor neurons and muscle fibers by intramuscular injection of HIV-1-based vectors pseudotyped with select rabies virus glycoproteins. J Neurosci Methods 157, 208–217. 16725205 10.1016/j.jneumeth.2006.04.011

[r24] Naldini, L., Blomer, U., Gallay, P., Ory, D., Mulligan, R., Gage, F. H., Verma, I. M. & Trono, D. (1996). *In vivo* gene delivery and stable transduction of nondividing cells by a lentiviral vector. Science 272, 263–267. 8602510 10.1126/science.272.5259.263

[r25] Op De Beeck, A., Voisset, C., Bartosch, B., Ciczora, Y., Cocquerel, L., Keck, Z., Foung, S., Cosset, F. L. & Dubuisson, J. (2004). Characterization of functional hepatitis C virus envelope glycoproteins. J Virol 78, 2994–3002. 14990718 10.1128/JVI.78.6.2994-3002.2004 PMC353750

[r26] Soda, Y., Shimizu, N., Jinno, A., Liu, H. Y., Kanbe, K., Kitamura, T. & Hoshino, H. (1999). Establishment of a new system for determination of coreceptor usages of HIV based on the human glioma NP-2 cell line. Biochem Biophys Res Commun 258, 313–321. 10329384 10.1006/bbrc.1999.0633

[r27] Stoker, M. & Macpherson, I. (1964). Syrian hamster fibroblast cell line BHK21 and its derivatives. Nature 203, 1355–1357. 14207308 10.1038/2031355a0

[r28] Stratton, M. R., Darling, J., Pilkington, G. J., Lantos, P. L., Reeves, B. R. & Cooper, C. S. (1989). Characterization of the human cell line TE671. Carcinogenesis 10, 899–905. 2650908 10.1093/carcin/10.5.899

[r29] Takeuchi, Y., Porter, C. D., Strahan, K. M., Preece, A. F., Gustafsson, K., Cosset, F. L., Weiss, R. A. & Collins, M. K. (1996). Sensitization of cells and retroviruses to human serum by (alpha 1–3) galactosyltransferase. Nature 379, 85–88. 8538747 10.1038/379085a0

[r30] Temperton, N. J., Chan, P. K., Simmons, G., Zambon, M. C., Tedder, R. S., Takeuchi, Y. & Weiss, R. A. (2005). Longitudinally profiling neutralizing antibody response to SARS coronavirus with pseudotypes. Emerg Infect Dis 11, 411–416. 15757556 10.3201/eid1103.040906 PMC3298259

[r31] Temperton, N. J., Hoschler, K., Major, D., Nicolson, C., Manvell, R., Hien, V. M., Ha, D. Q., Jong, M. D., Zambon, M. & other authors (2007). A sensitive retroviral pseudotype assay for influenza H5N1-neutralizing antibodies. Influenza Resp Viruses 1, 105–112. 10.1111/j.1750-2659.2007.00016.x PMC4941878 19453415

[r32] Towers, G. J. (2007). The control of viral infection by tripartite motif proteins and cyclophilin A. Retrovirology 4, 40 17565686 10.1186/1742-4690-4-40 PMC1906832

[r33] Towers, G., Bock, M., Martin, S., Takeuchi, Y., Stoye, J. P. & Danos, O. (2000). A conserved mechanism of retrovirus restriction in mammals. Proc Natl Acad Sci U S A 97, 12295–12299. 11027299 10.1073/pnas.200286297 PMC17335

[r34] WHO (2006). *Rabies*. Fact Sheet No. 99. Geneva, World Helath Organisation.

[r35] Wool-Lewis, R. J. & Bates, P. (1998). Characterization of Ebola virus entry by using pseudotyped viruses: identification of receptor-deficient cell lines. J Virol 72, 3155–3160. 9525641 10.1128/jvi.72.4.3155-3160.1998 PMC109772

[r36] Zufferey, R., Nagy, D., Mandel, R. J., Naldini, L. & Trono, D. (1997). Multiply attenuated lentiviral vector achieves efficient gene delivery in vivo. Nat Biotechnol 15, 871–875. 9306402 10.1038/nbt0997-871

