# Supplementary material for: Investigating antibody neutralization of lyssaviruses using lentiviral pseudotypes: a cross-species comparison
Source: J Gen Virol. 2008 Sep;89(Pt 9):2204–13. doi: 10.1099/vir.0.2008/000349-0 (PMC2886951; doi:10.1099/vir.0.2008/000349-0)
Supplement: [Supplementary Material] [file supp_89_9_2204__index.html]

 Investigating antibody neutralization of lyssaviruses using lentiviral pseudotypes: a cross-species comparison -- Wright et al. 89 (9): 2204 Data Supplement - Supplementary Material -- Journal of General Virology

## Supplementary Material

### Investigating antibody neutralization of lyssaviruses using lentiviral pseudotypes: a cross-species comparison, by E. Wright, N. J. Temperton, D. A. Marston, L. M. McElhinney, A. R. Fooks and R. A. Weiss

*Journal of General Virology* vol. **89**, part 9, pp. 2204 - 2213

**Supplementary Fig. S1.** Stability of lyssavirus pseudotypes.

**Supplementary Table S1.** Primers used to amplify G-protein sequences.

**Supplementary Table S2.** Comprehensive details of sera used in this study.

Single document. [PDF] (68 kb)

  
  
