# Supplementary material for: Investigating antibody neutralization of lyssaviruses using lentiviral pseudotypes: a cross-species comparison
Source: J Gen Virol. 2008 Sep;89(Pt 9):2204–13. doi: 10.1099/vir.0.2008/000349-0 (PMC2886951; doi:10.1099/vir.0.2008/000349-0)
Supplement: [Supplementary Material] [file supp_89_9_2204__1.pdf]

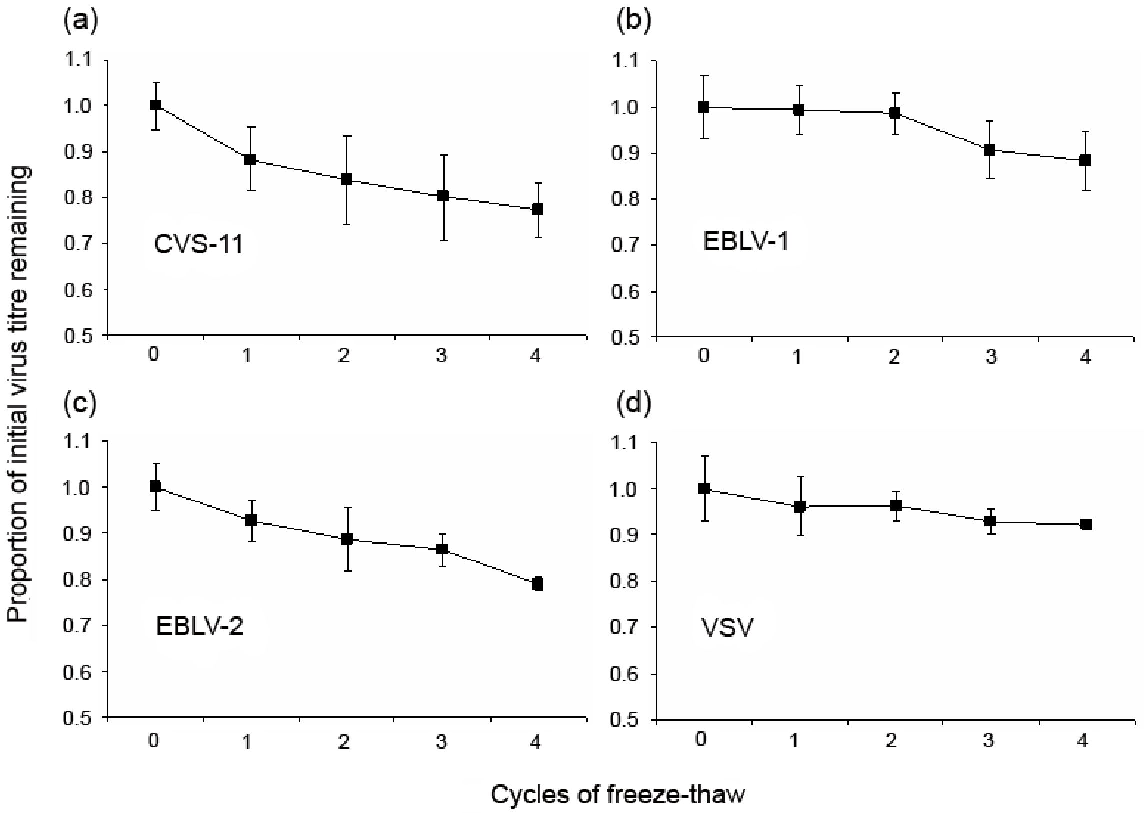

Fig. S1. Stability of lyssavirus pseudotypes. Lentiviral pseudotypes packaging the luciferase reporter and expressing (a) CVS-11, (b) EBLV-1, (c) EBLV-2 and (d) VSV G-proteins were subjected to four cycles of freeze–thaw. A 5 µl aliquot of each sample was then incubated with 1×10^4 BHK cells for 48 h, after which the level of viral infection was determined; this is reported as the proportion of virus remaining compared to the sample that underwent no (0) freeze–thaw cycles. The mean±SD of four experiments is shown.

Wright, E., Temperton, N. J., Marston, D. A., McElhinney, L. M., Fooks, A. R. & Weiss, R. A. (2008). Investigating antibody neutralization of lyssaviruses using lentiviral pseudotypes: a cross-species comparison. *J Gen Vir* **89**, 2204–2213.

Table S1. Primers used to amplify G-protein sequences

Name	Sequence (5′–3′)
CVS-11F	GCGCGCGGTACCGCCACCATGGTTCCTCAGGTTCTT
CVS-11R	GCGCGCCTCGAGTTACAGTCTGATCTCACCTC
EBLV-1F	GCGCGCGGTACCGCCACCATGTTACTCTCTACCGCCAT
EBLV-1R	GCGCGCCTCGAGTTATGACTCACCAGTGGC
EBLV-2F	GCGCGCGGTACCGCCACCATGCCATTCCAAGCTGTT
EBLV-2R	GCGCGCCTCGAGTTAAGACTGACCCCCCGTCT

Table S2. Comprehensive details of sera used in this study. The OIE standard reference dog serum and a commercially available negative control sera (Harlan Sera-Lab) were also used.

Serum ID	Species	Breed	Age*	Sex	Date of vaccination†	Date bled†	Vaccine	FAVN titre (IU ml^{-1})
PET-5825	Canine	Cross-breed	5 yrs	Female	18.04.07	13.06.07	Nobivac	0.87
PET-1333	Canine	Border Collie	3 yrs	Male	18.01.07	08.02.07	Nobivac	1.5
PET-1326	Canine	Staffordshire Bull Terrier	4 mths	Female	04.01.07	05.02.07	Nobivac	4.5
PET-1329	Canine	Boxer	4 yrs	Female	08.01.07	08.02.07	Nobivac	10.26
RS 175/07	Human	‡	38 yrs	Male	‡	15.08.07	‡	23.38
RC 199/07	Canine	Pug	3 yrs	Female	‡	25.07.07	‡	23.38
PET-1335	Feline	DSH§	1 yr	Female	18.10.07	01.02.07	Virbac	23.38
RC 248/07	Feline	DSH§	3 yrs	Female	18.07.07	17.08.07	Virbac	30.77
RC200/07	Canine	Mixed	1 yr	Female	‡	25.06.07	‡	30.77
2857/06	Human	‡	28 yrs	Male	‡	15/09/2007	‡	53.0
RS 174/07	Human	‡	20 yrs	Male	‡	15.08.07	‡	121.5
RC 215/07	Canine	Cross-breed	5 yrs	Female	08.05.07	30.06.07	Eurican	210.44
RC 195/07	Feline	DSH§	2 yrs	Female	26.12.06	20.06.07	Nobivac	364.5
PET-1310	Feline	DSH§	16 yrs	Female	27.01.07	07.02.07	Nobivac	631.33
5517	Canine	Mongrel	5 yrs	Male	27.04.07	08.06.07	Nobivac	0.29
5531	Canine	Labrador	1 yr	Female	14.05.07	13.06.07	Nobivac	0.38
5545	Canine	Rhodesian Ridgeback	6.5 yrs	Male	14.05.07	14.06.07	Rabisin	0.22
5546	Feline	‡	8 yrs	Male	23.05.07	13.06.07	Nobivac	0.38

5610	Feline	DSH§	11 yrs	Male	17.05.07	18.06.07	Rabisin	0.38
5637	Canine	Newfoundland	4 mths	Male	17.05.07	18.06.07	Rabisin	0.13
5710	Canine	Staffordshire Bull Terrier	4 mths	Female	21.05.07	20.06.07	Nobivac	0.06
5734	Canine	Border Collie	8 years	Male	01.06.07	22.06.07	Rabisin	0.38
5775	Canine	Rottweiler	15 wks	Male	31.05.07	21.06.07	Nobivac	0.29
5791	Canine	Cross-breed	3 yrs	Male	08.05.07	13.06.07	Virbagen	0.06
5858	Canine	Bedlington Terrier	9.5 yrs	Male	16.11.06	26.06.07	Rabisin	0.1
5890	Canine	Flat Coat Retriever	5 yrs	Male	11.05.07	27.06.07	Nobivac	0.06
5894	Canine	German Shepard	9 yrs	Female	11.06.07	25.06.07	Quantum	0.04
5896	Canine	Jack Russell Terrier	11 yrs	Male	11.06.07	25.06.07	Quantum	0.07
5910	Canine	Cross-breed	18 mths	Male	18.05.07	28.06.07	Quantum	0.1
5927	Feline	DSH§	12 yrs	Male	25.05.07	27.06.07	Nobivac	0.1
5930	Canine	King Charles Spaniel	4 mths	Male	06.06.07	27.06.07	Nobivac	0.29
5950	Canine	Bernese Mountain Dog	7 yrs	Male	31.05.07	27.06.07	Nobivac	0.22
5977	Canine	Golden Retriever	1 yr	Male	26.02.07	24.06.07	Fort Dodge	0.22
6041	Canine	German Shepherd	3 yrs	Female	06.06.07	03.07.07	Nobivac	0.29
6048	Canine	Cross-breed	2 yrs	Male	18.06.07	02.07.07	Rabisin	0.1
6108	Canine	Labrador Retriever	5 yrs	Female	29.05.07	29.06.07	Nobivac	0.38
6115	Canine	Retriever	8 yrs	Female	11.06.07	02.07.07	Rabisin	0.04
6126	Canine	Boxer	3 mths	Female	12.06.07	03.07.07	Nobivac	0.38
6144	Canine	Border Collie	9 yrs	Male	08.06.07	04.07.07	Rabisin	0.04
6146	Canine	Labrador Retriever	6 yrs	Male	25.05.07	04.07.07	Nobivac	0.29
6183	Feline	DSH§	15 yrs	Female	04.06.07	05.07.07	Nobivac	0.04

6203	Canine	Golden Retriever	10 mths	Male	14.05.07	06.07.07	Nobivac	0.38
6216	Canine	Labrador Retriever	8 yrs	Female	05.06.07	04.07.07	Quantum	0.29
6266	Canine	Dachshund	11 yrs	Male	05.06.07	09.07.07	Canigen	0.38
6271	Canine	Golden Retriever	1 yr	Male	07.07.07	07.07.07	Nobivac	0.17
6325	Feline	‡	8 mths	Male	27.02.07	08.07.07	Nobivac	0.38
6360	Canine	Scottish Terrier	6 yrs	Male	01.06.07	10.07.07	Nobivac	0.1
6369	Canine	West H.W. Terrier	10 yrs	Female	18.01.07	10.07.07	Nobivac	0.06
6374	Canine	Cross-breed	5 yrs	Male	20.06.07	11.06.07	Nobivac	0.38
6386	Canine	Boxer	6 mths	Female	16.04.07	01.07.07	Fort Dodge	0.17
6389	Canine	Labrador Retriever	6.5 yrs	Male	21.03.07	02.07.07	Nobivac	0.07
6460	Canine	Golden Retriever	8 yrs	Female	11.06.07	16.07.07	Rabisin	0.07
6507	Canine	Scottish Terrier	8 yrs	Female	08.06.07	17.07.07	Nobivac	0.17
6533	Canine	French Bulldog	5 mths	Male	22.05.07	17.07.07	Canigen	0.38
6579	Canine	Boxer	8 yrs	Male	04.06.07	19.07.07	Rabisin	0.29
6602	Canine	Springer Spaniel	2 yrs	Female	18.04.07	19.07.07	Rabisin	0.22
6604	Canine	German Shepherd	2 yrs	Female	18.01.07	12.07.07	Nobivac	0.22
6698	Canine	Cross-breed	12 yrs	Male	13.06.07	16.07.07	Rabisin	0.29

*, Age is given in weeks (wks), months (mths) or years (yrs).

†, Dates are given as DD.MM.YY.

‡, Data unavailable.

§, Feline breed DSH is domestic short hair.

Wright, E., Temperton, N. J., Marston, D. A., McElhinney, L. M., Fooks, A. R. & Weiss, R. A. (2008). Investigating antibody neutralization of lyssaviruses using lentiviral pseudotypes: a cross-species comparison. *J Gen Vir* **89**, 2204–2213.
